# Deep breathing exercise at work: Potential applications and impact

**DOI:** 10.3389/fphys.2023.1040091

**Published:** 2023-01-12

**Authors:** Dallin Tavoian, Daniel H. Craighead

**Affiliations:** ^1^ Arizona Respiratory Neurophysiology Laboratory, Department of Physiology, University of Arizona College of Medicine, Tucson, AZ, United States; ^2^ Integrative Physiology of Aging Laboratory, Department of Integrative Physiology, University of Colorado Boulder, Boulder, CO, United States

**Keywords:** hypertension, diaphragmatic breathing, stress, workplace, blood pressure

## Abstract

Hypertension is a major contributor to cardiovascular disease and daily deep breathing exercise (DBE) is a promising intervention to reduce blood pressure and stress in adults. DBE is simple, time-efficient, and does not require specialized equipment, allowing participation in a wide variety of settings. The workplace is an ideal setting to implement DBE at the national level for several reasons, including a large proportion of waking hours spent in the workplace, high levels of sedentary time at work, prevalence of work-related stress, and regular breaks throughout the day potentially reducing worker error. While the degree of adherence to daily workplace DBE will be the responsibility of the individual, employers and managers can (and should) do much to remove barriers to participation. Specifically, this could include: implementing regular short breaks or classes to perform DBE throughout the day, covering subscription costs for smartphone applications that guide DBE, and creating incentive programs for continuing DBE participation. Implementing DBE in the workplace is a pragmatic approach to provide a low-cost blood pressure and stress reduction therapy to a substantial portion of the adult population in the US, at least 50% of whom have high blood pressure.

## Introduction

Daily deep breathing exercise (DBE) can lower resting blood pressure (BP) and reduce stress and anxiety (Russo et al., 2017; Zaccaro et al., 2018; Yau and Loke, 2021). Further, as an exercise that can be performed virtually anywhere without any equipment required, there are few barriers to DBE, relative to traditional exercise strategies. The workplace serves as a source of stress for many adults, while simultaneously resulting in long periods of sedentary time for a large portion of the US workforce in our increasingly automated society. Chronic stress and high levels of sedentary time contribute directly to the development and worsening of cardiovascular disease (CVD) (Diaz et al., 2017; Kivimäki and Steptoe, 2018), the leading cause of death throughout the world for the last two decades (World Health Organization, 2020). Further, CVD-related disability negatively impacts workplace productivity (Virtanen et al., 2017). Above-normal BP (i.e., ≥120/80 mmHg) is a primary modifiable risk factor for CVD (Stanaway et al., 2018), and adults with above-normal BP face annual healthcare costs that are approximately $2,000 higher on average than normotensive adults (Kirkland et al., 2018), making BP reduction a key therapeutic target. Regular exercise participation, along with other lifestyle changes (e.g., diet), is recommended to maintain optimal health as well as counter CVD progression and lower BP (Li et al., 2018). Unfortunately, less than half of US adults exercise regularly (NCHS. National Center for Health Statistics, 2017); a trend that will likely continue despite decades of national programs/initiatives to increase physical activity levels. As such, the workplace is an ideal location to implement daily DBE, both as an additive strategy for those who exercise regularly and as a gateway exercise for those who do not exercise regularly. Employers are partially responsible for their employee’s job satisfaction and wellbeing, and thus could provide resources and regular breaks for DBE to improve these outcomes. In this Perspective article we will provide a brief overview of the key physiological and psychological benefits of DBE. We will then discuss the impact of CVD and stress on workplace productivity, and the potential for DBE to reduce CVD risk, as well as acute and chronic stress. Finally, we will discuss real-world application potential of DBE in the workplace, as well as current research gaps and future directions. Our overall goal is to highlight the potential health and productivity benefits of DBE and call for additional investigations surrounding this promising lifestyle intervention. In-depth reviews of the underlying mechanisms leading to the changes discussed herein are outside the scope of this manuscript, but can be found elsewhere (Russo et al., 2017; Zaccaro et al., 2018; Yau and Loke, 2021).

## Deep breathing exercise

**FIGURE 1:**
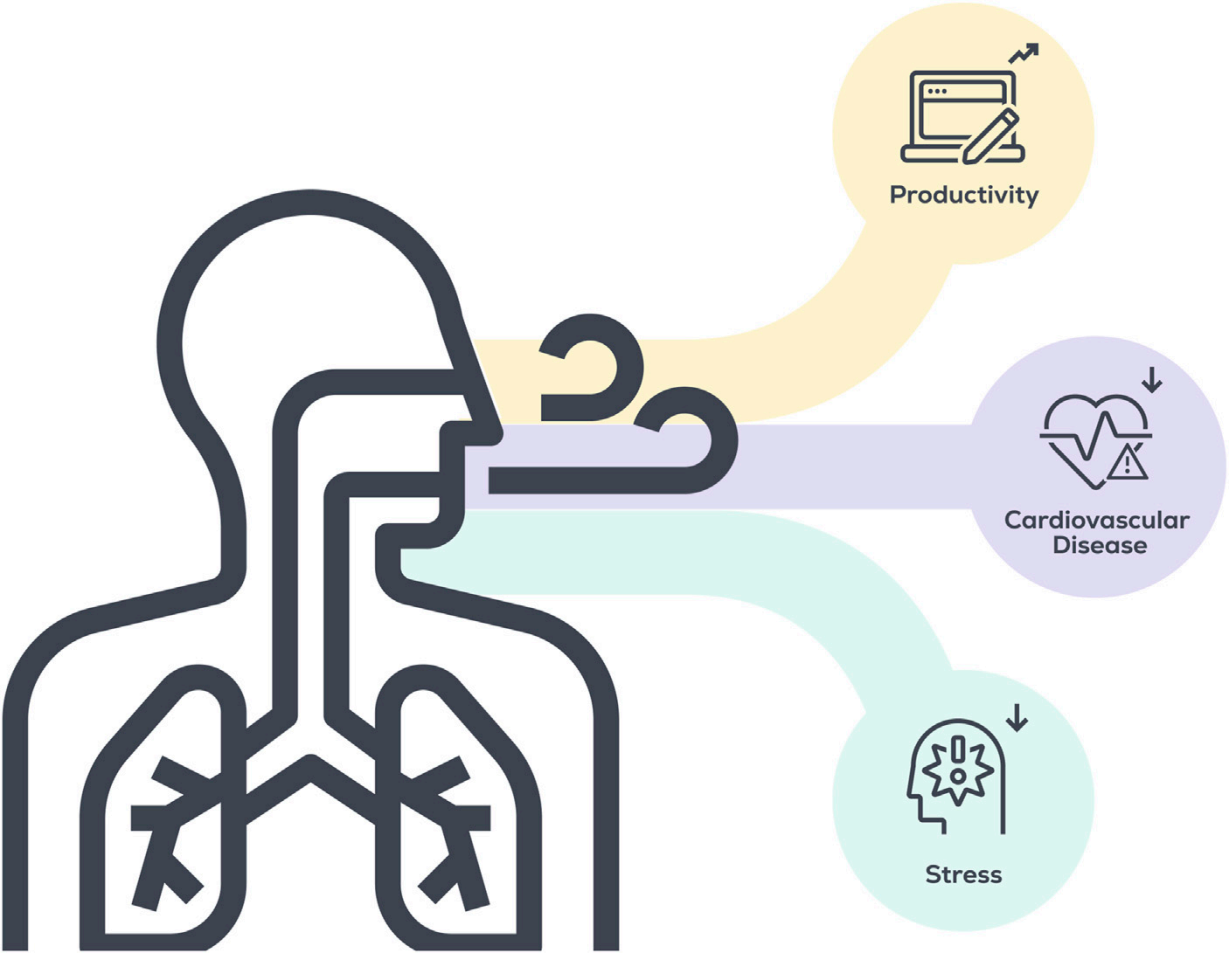
Potential benefits of implementing DBE in the workplace.

DBE is a broad term that encompasses several types of non-resisted, paced breathing strategies, including yogic breathing or Pranayama, diaphragmatic breathing, and abdominal breathing, to name a few (Bruton et al., 2011; Jayawardena et al., 2020). Along with their being differences between overall types of breathing training, there also exist significant differences in the specifics of how breathing training is employed. For example, two studies purporting to investigate “diaphragmatic breathing” may differ in the employed breathing frequency (breaths/minute), the fraction time spent inhaling and exhaling per breath, the utilization of nose or mouth breathing, the amount of time spent per day performing DBE, or the time of day that the intervention is performed, along with many other variables (Bruton et al., 2011). Given the number of variables at play, it is not surprising that current studies investigating breathing interventions have employed a wide degree of study designs, making it challenging to fully understand the potential health benefits of breathing training at the current moment. However, the preponderance of evidence does suggest DBE can improve certain important aspects of human health, including reduced BP and psychological stress (Zaccaro et al., 2018; Chaddha et al., 2019) (Figure 1), which may indirectly increase productivity through less utilization of sick time. Further, common barriers to traditional exercise strategies in non-exercising adults (e.g., lack of energy, poor health/physical disabilities, equipment/facilities access, work commitments/lack of time, cost, fear of pain/discomfort, bad weather, lack of knowledge, and low motivation (Booth, Bauman, Owen, Gore, 1997; Zunft et al., 1999; Baillot et al., 2021; Gee et al., 2012)) are minimized by DBE, and providing time and/or space for DBE in the workplace will help to overcome many of the remaining barriers. However, lack of knowledge and low motivation are two interconnected hurdles that must be addressed prior to widespread participation. Potential solutions to these barriers will be discussed in later sections.

## Physiological effects of deep breathing exercise

Clinical efficacy studies (i.e., those performed in tightly controlled laboratory conditions) suggest breathing training can lower BP. Two meta-analyses encompassing 22 randomized controlled trials (median intervention duration 8 weeks) and 1,344 participants with CVD and/or above-normal BP reported a 6 mmHg reduction in systolic BP and a 3–6 mmHg reduction in diastolic BP, on average (Zou et al., 2017; Chaddha et al., 2019). Importantly, these reductions are similar to those seen in response to other non-pharmacological lifestyle interventions (e.g., DASH diet, sodium restriction, caloric restriction, aerobic exercise, meditation (Fu et al., 2020)). Further, a large multi-center study (21,563 participants) reported that a single bout of DBE (6 breaths over 30 s) acutely reduced systolic BP 3–4 mmHg and diastolic BP 1 mmHg, relative to a time-matched rest interval (Mori et al., 2005). However, BP reduction in response to DBE is not a universal finding, as some reviews of the literature, specifically those quantifying the effects of device-guided DBE (i.e., RESPeRATE device), have found no benefit of DBE on BP compared to active control treatments (Landman et al., 2014; Freitas Gonçalves et al., 2022). Despite these reports, a preponderance of the evidence suggests that breathing interventions, primarily slow deep breathing, can lower BP, though the extent of the reduction likely varies considerably based on intervention designs. The reductions in systolic BP (i.e., 3–6 mmHg) observed following breathing training are likely to be clinically significant, as reductions of 5 mmHg achieved with pharmacotherapy are associated with a 10% lower risk for CVD (Blood Pressure Lowering Treatment Trialists’ Collaboration, 2021), while other studies suggest that even smaller reductions (i.e., 2 mmHg) can decrease risk for stroke and heart disease by 7%–10% (Lewington et al., 2002). Additional physiological adaptations to DBE include alterations in autonomic activity, increased heart rate variability, augmentation of baroreflex sensitivity, and improved ventilation efficiency (Russo et al., 2017; Zaccaro et al., 2018; Yau and Loke, 2021). There is also limited evidence that these physiological adaptations have a beneficial effect on some common health disorders, including gastrointestinal disorders, migraines, chronic obstructive pulmonary disease, and asthma (Hamasaki, 2020), and may improve sleep quality when performed prior to going to bed (Tsai et al., 2015; Laborde et al., 2019; Kuula et al., 2020), though these investigations are few and have notable limitations.

## Psychological effects of deep breathing exercise

Acutely, breathing interventions appear effective for reducing feelings of anxiety or depression (Zaccaro et al., 2018; Hopper et al., 2019; Yau and Loke, 2021), and DBE is a reliable method of controlling panic attacks by countering hyperventilation, a common symptom in adults with panic disorder (Cowley and Roy-Byrne, 1987). A single bout of DBE (5–20 min) reportedly reduces subjective feelings of anxiety in generally healthy young and older adults (Yu et al., 2011; Magnon et al., 2021) and in men with alcohol dependence (Clark and Hirschman, 1990), while also reducing feelings of depression and anger-hostility in healthy adults (Yu et al., 2011). In regard to long-term benefits, evidence from clinical trials support the chronic effects of breathing interventions for improving mental health parameters as well. For example, 20 sessions of 15 min of diaphragmatic breathing over 8 weeks reduced negative affect (i.e., negative emotions and expression) and physiological markers of stress (i.e., salivary cortisol), but did not alter positive affect (i.e., positive emotions and expression) in healthy adults (Ma et al., 2017). Other 8-week randomized controlled trials in adults with anxiety (Chen et al., 2017) or major depressive disorder (Sharma et al., 2017) demonstrated the effectiveness of DBE for improving self-reported anxiety and indices of depression, respectively, though it should be noted that in adults with depression, the experimental group performed yoga poses in addition to modified breathing (Sharma et al., 2017). In a separate non-randomized trial, those encouraged to perform 10 min of deep breathing, twice per day, for 9 months demonstrated greater reductions in self-reported stress than those in the control group (Sundram et al., 2014). Chronic stress/anxiety is an independent risk factor for hypertension (Bhelkar et al., 2018) and is associated with increased sympathetic activity (Won and Kim, 2016), and it is possible that these physiological adaptations are simply a side effect of the reduced stress seen in response to DBE.

## Cardiovascular disease and workplace stress

CVD has been the leading cause of death in the US for the last two decades (World Health Organization, 2020). Further, those living with CVD are likely to develop additional chronic diseases and early onset physical disability (Jin et al., 2019; Vassalle et al., 2022). CVD is prevalent in middle-age US adults, and therefore imposes substantial morbidity/mortality-related productivity costs in the workplace, including greater utilization of sick time for longer periods and time off work to recover from cardiac events (Virtanen et al., 2017). Both chronic stress and depression independently increase CVD risk (Strike and Steptoe, 2002; Kivimäki and Steptoe, 2018), and the workplace is a primary source of stress for many adults, with 85% of respondents to a 2021 survey indicating that workplace stress affects their mental health (Mental Health America, 2021), while a separate report found that 76% of working US adults reported experiencing at least one symptom of a mental health condition (i.e., burnout, depression, anxiety) due to work in the last year (Partners, 2021). One in two US adults have high BP (Whelton et al., 2018) and those adults who work ≥49 h/week have a 70% greater chance of developing hypertension (Trudel et al., 2020). Employees with chronic work stress have a 50% excess risk for coronary heart disease than those without work stress (Kivimäki et al., 2006), and acute stress events can trigger cardiac events in those with ongoing CVD, whereas chronic stress is associated with recurrent cardiac events (Steptoe and Kivimäki, 2012). As a result, a record number of adults are leaving their jobs in what is coming to be known as the “Great Resignation”, with at least half of adults who left their jobs citing mental health reasons (Partners, 2021) and ∼40% reporting feeling disrespected at work (Pew Research Center, 2022). The workplace has transformed from a strictly professional setting to a vibrant social scene where people want to connect with their coworkers and enjoy their time. If employees feel negatively about their work situation (e.g., unsupported by management, lack of growth potential, disrespect) they will find an alternative job that evokes positive feelings, or one with sufficient monetary compensation to overcome the negative aspects. It has become the responsibility of the employer to provide a working environment that is supportive for employees; a place where they want to spend a large portion of their time every day. Several private companies understand this and have invested in perks for employees, including exercise equipment on site, mental health resources, free subscriptions to mindfulness apps, and healthy food alternatives, to name a few (Glassdoor, 2019).

It is worth noting that not all individuals perceive stress in the same way and that the workplace may not necessarily be the underlying cause of stress for all people. Stress and the stress response are multifactorial processes, influenced by early life experiences, race, gender identity, socioeconomic status, and personality type, to name a few (Williams, 1999; Vollrath, 2001; LeMoult et al., 2020; Tan et al., 2020). Further, there may be workplace stressors that are too severe to be resolved with DBE (e.g., workplace harassment). With this in mind, DBE should not be seen as a replacement for established pharmacological or behavioral therapies to treat stress, anxiety, or depression. Instead, we recommend DBE be used as a stress-reduction tool to supplement any ongoing or planned mental health treatment.

## Real-world application

At least two studies assessing the effectiveness of DBE in the workplace have been published to date (Alexopoulos et al., 2014; Sundram et al., 2014), neither of which reported sufficient methodological details (e.g., breathing rate) to replicate the intervention. However, what we can pull from these investigations is that lack of supervisor support and high work-load are major barriers to regular DBE participation (Sundram et al., 2014). Further, DBE can easily be paired with other relaxation activities (e.g., mindfulness, progressive muscle relaxation) as they can be performed simultaneously (Alexopoulos et al., 2014).

There is a wealth of DBE resources online and in written format. There are essentially limitless combinations of breathing factors (e.g., inhale/exhale ratios, nose or mouth breathing, exercise duration) that could be employed, yet not enough evidence to recommend one protocol over another. However, the two components of DBE that appear to be most consistent among protocols are 1) depth of breath (i.e., at least 80% of vital capacity) and 2) rate of breathing (i.e., approximately 0.1 Hz or slower, or about 6 breaths/minute or fewer). Thus, if these criteria are met, the other aspects of the protocol may become less important. Given the above, two important public health goals become 1) increasing DBE adoption on a national or international scale; and 2) promoting long-term adherence to DBE by new and current participants by removing as many barriers to DBE as possible.

As mentioned previously, there are several barriers to traditional exercise strategies that do not necessarily pertain to DBE. However, relevant barriers to DBE include lack of knowledge, low motivation, and lack of support. In fact, because of the simplicity of DBE, successful adherence strategies probably closely reflect the strategies used to increase adherence to medications, including increasing patient self-efficacy, belief in the effectiveness of the treatment, and social support (Holmes et al., 2014). Additional medication adherence strategies like having an established routine and cues as reminders (Chambers et al., 2011) (e.g., hourly smartwatch notifications) would likely be effective for DBE as well.

The workplace is an ideal venue to implement DBE at a large scale, however, there are some key considerations that employers should keep in mind. A recent qualitative analysis directed toward understanding the psychological and social influences surrounding taking breaks at work has identified some key themes that can provide insight to employers (Oliver et al., 2021). With these considerations in mind, we have created some recommendations on how to promote daily DBE at work: 1) regular DBE breaks throughout the day must become part of the culture at work and employers/manager should lead by example, 2) employers attitudes toward breaks must be perceived as positive by employees and there should not be pressure from management to work during breaks, 3) it must be clear that these short breaks are added on top of breaks already in place (e.g., lunch break) and not replacing them, 4) employees should feel that their physical and mental health is valued more than their productivity, and 5) break time should be protected (those practicing DBE should not be interrupted for work responsibilities until break time is over). While there may be concerns that frequent breaks will reduce productivity, current evidence suggests no negative effects of short, regular breaks on productivity (Waongenngarm et al., 2018), and, in fact, they can reduce perceived tiredness, stress, and job errors (Mitra et al., 2008; Taylor, 2011; Randolph, 2016).

**FIGURE 2:**
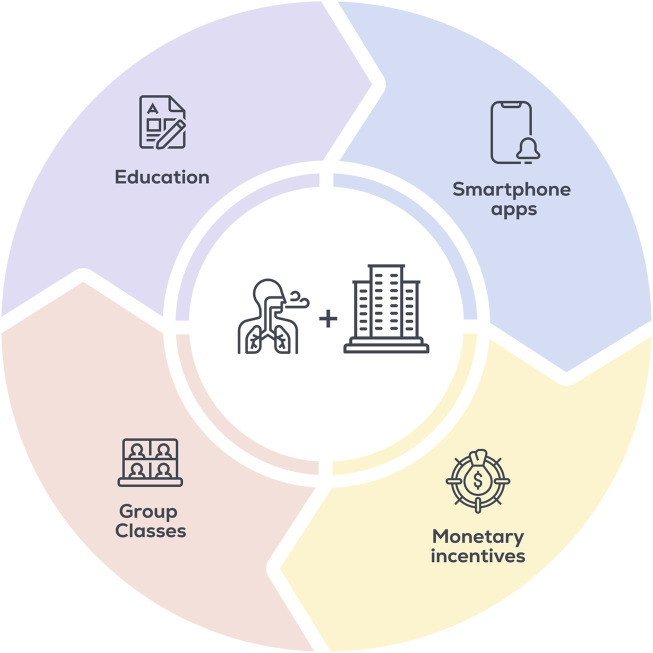
Strategies to implement DBE and increase adherence in the workplace.

Additional solutions employers could use to overcome barriers to regular DBE include providing educational resources regarding the beneficial effects of DBE, implementing in-person deep breathing “classes” that employees can attend throughout the day, covering subscription costs for smartphone applications that guide DBE (e.g., Breathwrk, Breathe+), and/or create incentive programs for continuing DBE participation (Figure 2). Smartphone applications have been shown to increase adherence to lifestyle interventions (Carter et al., 2013), and generally contain additional features to increase engagement (e.g., tracking, leaderboards, personalization, education). Further, these applications provide multiple types of guided or self-directed breathing protocols, allowing users to choose the type of DBE they prefer and find most effective at reducing stress for themselves. As of 2021, 85% of US adults reportedly own a smartphone (Pew Research Center, 2021), making application-directed DBE accessible to the majority of adults.

## Potential in other populations and future directions

While this Perspective article highlights the workplace as an ideal venue for large-scale DBE participation to impact physiological and psychological health at the national level, there are several other groups that could potentially benefit from regular DBE. High rates of acute and chronic stress/anxiety in adolescents (Merikangas et al., 2010) and young adults (Goodwin et al., 2020) highlight schools as a target for DBE participation. Similarly, individuals suffering from performance anxiety (e.g., athletes, musicians) could potentially benefit from DBE, and coaches/teachers could provide the resources suggested in this article. DBE may be particularly useful in unique clinical populations unable to perform traditional exercise strategies (e.g., individuals with orthopedic injuries, older adults with mobility limitations), as well as any population with above-normal BP (e.g., adults with obstructive sleep apnea). Indeed, there are several recently completed or ongoing clinical trials with the aim of validating DBE in distinct populations, including smokers (NCT03728530), women with pregnancy-induced hypertension (NCT04059822), adults with physical limitations (NCT05396027), post cardiac surgery patients (NCT01282671), and cancer patients undergoing radiotherapy (NCT04441827).

In addition to these disease/condition-specific investigations, larger and longer-term trials must be undertaken, ideally with adequate control groups that will help investigators to differentiate the effects of DBE from other stress-reducing therapies (e.g., mindfulness). Further, pragmatic experimental designs that take into account real-world barriers to habit forming and long-term adherence are warranted. DBE could also be compared against traditional exercise strategies or other interventions designed to decrease sedentary time. For example, whether DBE can counteract the negative effects of prolonged sitting has not been investigated to date. Recent reports indicate that just 5 min of walking, and even simply standing, every hour can counteract some of the negative effects of prolonged sitting (Benatti and Ried-Larsen, 2015; Chandrasekaran et al., 2021), and it is possible that DBE would result in similar beneficial effects. Ultimately, increased awareness and enhanced dissemination strategies will be necessary if wide-spread acceptance of DBE is to become a reality.

## Conclusion

Daily DBE can result in beneficial physiological adaptations, including reduced BP and sympathetic activity, and can reduce chronic stress and mitigate acute stress events. It should be noted that the evidence behind these assertions is somewhat limited. However, the simplicity of the technique, lack of negative effects, and high benefit/cost ratio (i.e., modest-moderate benefit with minimal cost/time commitment) make DBE a potential candidate for a large-scale intervention option targeted to individuals wherein BP and/or stress and anxiety could be reduced, and the workplace is an ideal setting to implement daily DBE. In contrast to traditional exercise strategies, DBE can be performed by any population and has few barriers to participation. Further, DBE can be performed essentially anywhere, has low energetic cost, and would have no negative effects on performance or productivity. The barriers that are inherent to habit-forming (e.g., lack of motivation) and thus regular DBE participation, could be mitigated by employers by providing resources for DBE and promoting a work culture that emphasizes the physical and mental health of its employees. Smartphone applications that guide users in DBE, as well as provide educational materials on the health benefits of DBE, may help to increase acceptance and adherence.

## Data Availability

The original contributions presented in the study are included in the article/supplementary material, further inquiries can be directed to the corresponding author.
